# Poly[diaqua-μ-oxalato-μ-pyrazine-2-carbox­yl­ato-lanthanum(III)]

**DOI:** 10.1107/S1600536808041664

**Published:** 2008-12-17

**Authors:** Lu Han, Qun-Hui Meng, Jian-Dong Hao, Yi-Fan Luo, Rong-Hua Zeng

**Affiliations:** a School of Chemistry and the Environment, South China Normal University, Guangzhou 510006, People’s Republic of China; b Key Laboratory of Technology on Electrochemical Energy Storage and Power Generation in Guangdong Universities, Guangzhou 510631, People’s Republic of China

## Abstract

In the title complex, [La(C_5_H_3_N_2_O_2_)(C_2_O_4_)(H_2_O)_2_]_*n*_, the La^III^ ion is coordinated by one N and three O atoms from two pyrazine-2-carboxylate ligands, by four O atoms from two oxalate ligands and by two O atoms of two water molecules, displaying a distorted bicapped square-anti­prismatic geometry. The carboxyl­ate groups of pyrazine-2-carboxyl­ate and oxalate ligands link the lanthanum metal centres, forming layers parallel to (10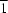). The layers are further connected by inter­molecular O—H⋯O and N—H⋯O hydrogen-bonding inter­actions, forming a three-dimensional supra­molecular network.

## Related literature

For general background, see: Eddaoudi *et al.* (2001 ▶); Rizk *et al.* (2005 ▶); Zeng *et al.* (2007 ▶). 
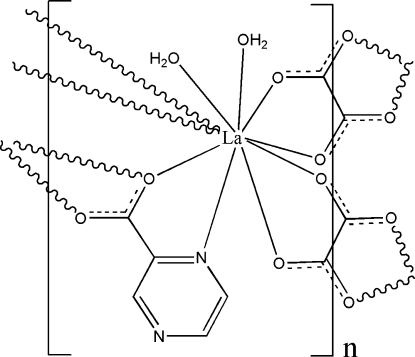


## Experimental

### 

#### Crystal data

[La(C_5_H_3_N_2_O_2_)(C_2_O_4_)(H_2_O)_2_]
*M* *_r_* = 386.06
Triclinic, 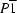

*a* = 8.040 (3) Å
*b* = 8.7343 (18) Å
*c* = 8.8329 (18) Å
α = 115.552 (2)°
β = 101.447 (3)°
γ = 95.789 (3)°
*V* = 536.1 (3) Å^3^
*Z* = 2
Mo *K*α radiation
μ = 4.02 mm^−1^
*T* = 296 (2) K
0.17 × 0.16 × 0.14 mm

#### Data collection

Bruker APEXII area-detector diffractometer
Absorption correction: multi-scan (*APEX2*; Bruker, 2004 ▶) *T*_min_ = 0.548, *T*_max_ = 0.603 (expected range = 0.518–0.569)
2761 measured reflections
1898 independent reflections
1787 reflections with *I* > 2σ(*I*)
*R*_int_ = 0.020

#### Refinement

*R*[*F* ^2^ > 2σ(*F* ^2^)] = 0.031
*wR*(*F* ^2^) = 0.078
*S* = 1.07
1898 reflections
163 parameters
6 restraints
H-atom parameters constrained
Δρ_max_ = 1.61 e Å^−3^
Δρ_min_ = −1.22 e Å^−3^

### 

Data collection: *APEX2* (Bruker, 2004 ▶); cell refinement: *SAINT* (Bruker, 2004 ▶); data reduction: *SAINT*; program(s) used to solve structure: *SHELXS97* (Sheldrick, 2008 ▶); program(s) used to refine structure: *SHELXL97* (Sheldrick, 2008 ▶); molecular graphics: *ORTEPIII* (Burnett & Johnson, 1996 ▶), *PLATON* (Spek, 2003 ▶) and *SHELXTL* (Sheldrick, 2008 ▶); software used to prepare material for publication: *SHELXL97*.

## Supplementary Material

Crystal structure: contains datablocks I, global. DOI: 10.1107/S1600536808041664/dn2413sup1.cif

Structure factors: contains datablocks I. DOI: 10.1107/S1600536808041664/dn2413Isup2.hkl

Additional supplementary materials:  crystallographic information; 3D view; checkCIF report

## Figures and Tables

**Table 1:** Hydrogen-bond geometry (Å, °)

*D*—H⋯*A*	*D*—H	H⋯*A*	*D*⋯*A*	*D*—H⋯*A*
O1*W*—H1*W*⋯N1^i^	0.84	1.97	2.796 (6)	170
O2*W*—H3*W*⋯O2^ii^	0.84	1.94	2.737 (5)	157
O1*W*—H2*W*⋯O3^iii^	0.84	2.05	2.874 (5)	167
O2*W*—H4*W*⋯O6^iv^	0.84	2.09	2.825 (5)	146

Symmetry codes: (i) 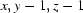; (ii) 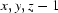; (iii) 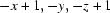; (iv) 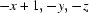.

**Fig. 1.**
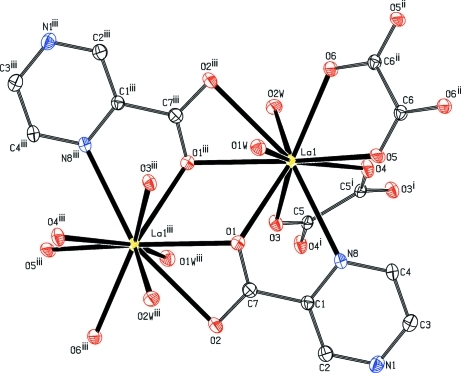
ORTEP view showing the atomic-numbering scheme. Displacement ellipsoids are drawn at the 30% probability level. [Symmetry codes: (i)1-x, 1-y, 1-z; (ii)1-x, -y, -z; (iii)1-x, -y, 1-z]

**Fig. 2.**
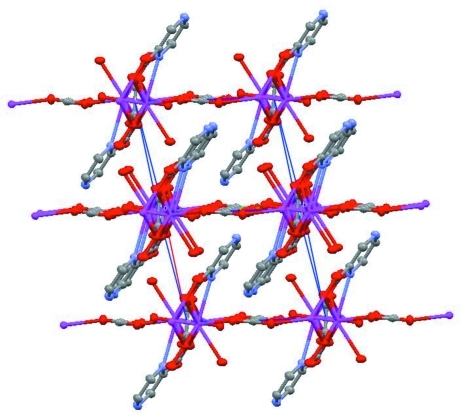
View of the layered network of the title structure.

## supplementary crystallographic information

### Comment

The design, synthesis, characterization, and properties of supramolecular networks formed by using functionalized organic molecules as bridges between metal centers are of great interest(Eddaoudi *et al.*, 2001; Rizk *et al.*, 2005; Zeng *et al.*,2007). As a building block, pyrazine-2-carboxylic acid and oxalic acid are excellent candidates for the construction of supramolecular complexes. Herein, we reported the new coordination polymer, (I).

In (I), each La^III^ centre is coordinated by seven oxygen atoms and one nitrogen atom from two pyrazine-2-carboxylate ligands, two oxalate ligands and two water molecules (Fig. 1), and represents a distorted bicapped square antiprismatic geometry. The La^III^ ions are linked by pyrazine-2-carboxylate ligands and oxalate ligands to form layers parallel to the (1 0 -1) plane (Fig.2), and the adjacent La···La separations are 6.570 (4) and 4.506 (5) Å, respectively. O—H···O and N—H···O hydrogen bonds (Table 1), involving the pyrazine-2-carboxylate ligands, coordinating water molecules and oxalate ligands assemble neighboring layers into a three-dimensional supramolecular network motif .

### Experimental

A mixture of La_2_O_3_ (0.245 g; 0.75 mmol), pyrazine-2-carboxylic acid (0.186 g; 1.5 mmol), oxalic acid(0.135 g; 1.5 mmol), water (10 mL) in the presence of HNO_3_ (0.024 g; 0.385 mmol) was stirred vigorously for 20 min and then sealed in a Teflon-lined stainless-steel autoclave (20 mL, capacity). The autoclave was heated and maintained at 433K for 3 days, and then cooled to room temperature at 5 K h^-1^ and obtained the colorless block crystals.

### Refinement

Water H atoms were tentatively located in difference Fourier maps and were refined with distance restraints of O–H = 0.84 Å and H···H = 1.35 Å, and with *U*_iso_(H) = 1.5 *U*_eq_(O). In the last cycles of refinement they were treated as riding on the O atoms. Carbon-bound H atoms were placed at calculated positions and were treated as riding on their parent C atoms with C—H = 0.93 Å, and with *U*_iso_(H) = 1.2 *U*_eq_(C).

### Crystal data

**Table:**

[La(C_5_H_3_N_2_O_2_)(C_2_O_4_)(H_2_O)_2_]	*Z* = 2
*M**_r_* = 386.06	*F*(000) = 368
Triclinic, *P*1	*D*_x_ = 2.391 Mg m^−^^3^
Hall symbol: -P 1	Mo *K*α radiation, λ = 0.71073 Å
*a* = 8.040 (3) Å	Cell parameters from 6377 reflections
*b* = 8.7343 (18) Å	θ = 1.7–28.0°
*c* = 8.8329 (18) Å	µ = 4.02 mm^−^^1^
α = 115.552 (2)°	*T* = 296 K
β = 101.447 (3)°	Block, colourless
γ = 95.789 (3)°	0.17 × 0.16 × 0.14 mm
*V* = 536.1 (3) Å^3^	

### Data collection

**Table:**

Bruker APEXII area-detector diffractometer	1898 independent reflections
Radiation source: fine-focus sealed tube	1787 reflections with *I* > 2σ(*I*)
graphite	*R*_int_ = 0.020
φ and ω scans	θ_max_ = 25.2°, θ_min_ = 2.6°
Absorption correction: multi-scan (*APEX2*; Bruker, 2004)	*h* = −5→9
*T*_min_ = 0.548, *T*_max_ = 0.603	*k* = −10→10
2761 measured reflections	*l* = −10→10

### Refinement

**Table:**

Refinement on *F*^2^	Primary atom site location: structure-invariant direct methods
Least-squares matrix: full	Secondary atom site location: difference Fourier map
*R*[*F*^2^ > 2σ(*F*^2^)] = 0.031	Hydrogen site location: inferred from neighbouring sites
*wR*(*F*^2^) = 0.078	H-atom parameters constrained
*S* = 1.07	*w* = 1/[σ^2^(*F*_o_^2^) + (0.0533*P*)^2^] where *P* = (*F*_o_^2^ + 2*F*_c_^2^)/3
1898 reflections	(Δ/σ)_max_ < 0.001
163 parameters	Δρ_max_ = 1.61 e Å^−^^3^
6 restraints	Δρ_min_ = −1.22 e Å^−^^3^

### Special details

**Table:**

Geometry. All esds (except the esd in the dihedral angle between two l.s. planes) are estimated using the full covariance matrix. The cell esds are taken into account individually in the estimation of esds in distances, angles and torsion angles; correlations between esds in cell parameters are only used when they are defined by crystal symmetry. An approximate (isotropic) treatment of cell esds is used for estimating esds involving l.s. planes.
Refinement. Refinement of F^2^ against ALL reflections. The weighted R-factor wR and goodness of fit S are based on F^2^, conventional R-factors R are based on F, with F set to zero for negative F^2^. The threshold expression of F^2^ > 2sigma(F^2^) is used only for calculating R-factors(gt) etc. and is not relevant to the choice of reflections for refinement. R-factors based on F^2^ are statistically about twice as large as those based on F, and R- factors based on ALL data will be even larger.

### Fractional atomic coordinates and isotropic or equivalent isotropic displacement parameters (Å^2^)

**Table:**

	*x*	*y*	*z*	*U*_iso_*/*U*_eq_	
C1	0.2750 (6)	0.2794 (6)	0.7523 (6)	0.0218 (10)	
C2	0.2577 (7)	0.4053 (7)	0.9066 (7)	0.0285 (11)	
H2	0.3191	0.4122	1.0111	0.034*	
C3	0.0676 (7)	0.4974 (7)	0.7564 (7)	0.0304 (12)	
H3	−0.0079	0.5706	0.7529	0.036*	
C4	0.0849 (7)	0.3716 (7)	0.6014 (7)	0.0289 (11)	
H4	0.0202	0.3621	0.4968	0.035*	
C5	0.5718 (6)	0.4928 (6)	0.5701 (6)	0.0204 (10)	
C6	0.0553 (6)	−0.0282 (6)	−0.0653 (6)	0.0218 (10)	
C7	0.3936 (6)	0.1564 (7)	0.7458 (6)	0.0238 (10)	
La1	0.33941 (3)	0.08521 (3)	0.32247 (3)	0.01745 (13)	
N1	0.1560 (6)	0.5173 (6)	0.9109 (6)	0.0284 (10)	
N8	0.1914 (5)	0.2634 (5)	0.5974 (5)	0.0235 (9)	
O1	0.3920 (5)	0.0398 (4)	0.5980 (4)	0.0248 (8)	
O2	0.4933 (5)	0.1760 (5)	0.8841 (5)	0.0314 (8)	
O3	0.5571 (5)	0.3495 (4)	0.5715 (4)	0.0246 (7)	
O4	0.6879 (4)	0.6241 (4)	0.6678 (4)	0.0264 (8)	
O5	−0.0155 (4)	−0.0721 (5)	−0.2201 (4)	0.0299 (8)	
O6	0.2119 (4)	−0.0240 (5)	−0.0025 (4)	0.0292 (8)	
O1W	0.2022 (5)	−0.2208 (4)	0.2499 (4)	0.0271 (8)	
O2W	0.5746 (5)	0.1980 (5)	0.2097 (4)	0.0300 (8)	
H1W	0.1827	−0.2913	0.1441	0.045*	
H3W	0.5293	0.2079	0.1210	0.045*	
H2W	0.2583	−0.2630	0.3071	0.045*	
H4W	0.6611	0.1534	0.1900	0.045*	

### Atomic displacement parameters (Å^2^)

**Table:**

	*U*^11^	*U*^22^	*U*^33^	*U*^12^	*U*^13^	*U*^23^
C1	0.018 (2)	0.027 (2)	0.021 (2)	0.007 (2)	0.005 (2)	0.012 (2)
C2	0.024 (3)	0.033 (3)	0.026 (3)	0.008 (2)	0.004 (2)	0.012 (2)
C3	0.030 (3)	0.030 (3)	0.033 (3)	0.012 (2)	0.010 (2)	0.014 (2)
C4	0.031 (3)	0.033 (3)	0.029 (3)	0.017 (2)	0.007 (2)	0.018 (2)
C5	0.015 (2)	0.025 (2)	0.021 (2)	0.0083 (19)	0.005 (2)	0.009 (2)
C6	0.017 (2)	0.023 (2)	0.024 (2)	0.0043 (19)	0.004 (2)	0.010 (2)
C7	0.018 (2)	0.033 (3)	0.025 (3)	0.005 (2)	0.007 (2)	0.016 (2)
La1	0.01352 (18)	0.01999 (18)	0.01806 (18)	0.00464 (11)	0.00163 (12)	0.00898 (13)
N1	0.022 (2)	0.027 (2)	0.031 (2)	0.0072 (18)	0.0067 (19)	0.0093 (19)
N8	0.022 (2)	0.027 (2)	0.023 (2)	0.0084 (17)	0.0035 (17)	0.0132 (18)
O1	0.0267 (19)	0.0270 (18)	0.0238 (18)	0.0104 (15)	0.0089 (15)	0.0126 (15)
O2	0.028 (2)	0.046 (2)	0.0244 (18)	0.0153 (17)	0.0049 (16)	0.0195 (17)
O3	0.0261 (19)	0.0225 (17)	0.0260 (18)	0.0047 (14)	0.0020 (15)	0.0143 (15)
O4	0.0196 (18)	0.0241 (18)	0.0316 (19)	0.0037 (15)	−0.0015 (15)	0.0131 (16)
O5	0.0172 (18)	0.049 (2)	0.0197 (18)	0.0097 (16)	0.0016 (15)	0.0132 (16)
O6	0.0147 (18)	0.046 (2)	0.0220 (17)	0.0100 (16)	0.0020 (14)	0.0123 (16)
O1W	0.0262 (19)	0.0239 (18)	0.0263 (18)	0.0031 (15)	0.0015 (15)	0.0100 (15)
O2W	0.027 (2)	0.039 (2)	0.0272 (19)	0.0109 (17)	0.0114 (16)	0.0166 (17)

### Geometric parameters (Å, °)

**Table:**

C1—N8	1.340 (6)	C7—O1	1.259 (6)
C1—C2	1.378 (7)	La1—O1W	2.533 (3)
C1—C7	1.497 (7)	La1—O4^i^	2.536 (3)
C2—N1	1.330 (7)	La1—O6	2.544 (3)
C2—H2	0.9300	La1—O3	2.551 (3)
C3—N1	1.336 (7)	La1—O5^ii^	2.555 (4)
C3—C4	1.382 (7)	La1—O1	2.592 (3)
C3—H3	0.9300	La1—O2W	2.600 (4)
C4—N8	1.332 (7)	La1—O1^iii^	2.623 (3)
C4—H4	0.9300	La1—N8	2.828 (4)
C5—O4	1.242 (6)	La1—O2^iii^	2.889 (4)
C5—O3	1.250 (6)	La1—C7^iii^	3.124 (5)
C5—C5^i^	1.574 (9)	O1W—H1W	0.8385
C6—O5	1.239 (6)	O1W—H2W	0.8353
C6—O6	1.261 (6)	O2W—H3W	0.8400
C6—C6^ii^	1.539 (9)	O2W—H4W	0.8421
C7—O2	1.252 (6)		
			
N8—C1—C2	122.0 (5)	O5^ii^—La1—O1^iii^	154.21 (12)
N8—C1—C7	115.3 (4)	O1—La1—O1^iii^	60.45 (13)
C2—C1—C7	122.7 (5)	O2W—La1—O1^iii^	75.58 (11)
N1—C2—C1	122.1 (5)	O1W—La1—N8	97.55 (12)
N1—C2—H2	118.9	O4^i^—La1—N8	72.37 (12)
C1—C2—H2	118.9	O6—La1—N8	128.17 (11)
N1—C3—C4	122.0 (5)	O3—La1—N8	68.57 (12)
N1—C3—H3	119.0	O5^ii^—La1—N8	66.44 (11)
C4—C3—H3	119.0	O1—La1—N8	58.52 (11)
N8—C4—C3	121.9 (5)	O2W—La1—N8	131.02 (12)
N8—C4—H4	119.0	O1^iii^—La1—N8	115.86 (11)
C3—C4—H4	119.0	O1W—La1—O2^iii^	66.41 (11)
O4—C5—O3	126.6 (4)	O4^i^—La1—O2^iii^	129.49 (11)
O4—C5—C5^i^	117.0 (5)	O6—La1—O2^iii^	68.05 (11)
O3—C5—C5^i^	116.4 (5)	O3—La1—O2^iii^	112.40 (11)
O5—C6—O6	126.1 (4)	O5^ii^—La1—O2^iii^	121.50 (11)
O5—C6—C6^ii^	118.0 (5)	O1—La1—O2^iii^	99.45 (10)
O6—C6—C6^ii^	115.9 (5)	O2W—La1—O2^iii^	65.10 (11)
O2—C7—O1	122.7 (5)	O1^iii^—La1—O2^iii^	46.83 (10)
O2—C7—C1	119.7 (5)	N8—La1—O2^iii^	157.23 (11)
O1—C7—C1	117.5 (4)	O1W—La1—C7^iii^	68.77 (12)
O2—C7—La1^iii^	67.6 (3)	O4^i^—La1—C7^iii^	137.10 (12)
O1—C7—La1^iii^	55.5 (3)	O6—La1—C7^iii^	91.66 (12)
C1—C7—La1^iii^	169.0 (3)	O3—La1—C7^iii^	95.00 (12)
O1W—La1—O4^i^	150.33 (12)	O5^ii^—La1—C7^iii^	139.98 (12)
O1W—La1—O6	82.82 (11)	O1—La1—C7^iii^	78.74 (11)
O4^i^—La1—O6	82.26 (12)	O2W—La1—C7^iii^	70.04 (12)
O1W—La1—O3	139.37 (10)	O1^iii^—La1—C7^iii^	23.29 (11)
O4^i^—La1—O3	63.95 (11)	N8—La1—C7^iii^	136.97 (12)
O6—La1—O3	136.33 (11)	O2^iii^—La1—C7^iii^	23.62 (11)
O1W—La1—O5^ii^	77.14 (12)	C2—N1—C3	116.0 (5)
O4^i^—La1—O5^ii^	73.25 (12)	C4—N8—C1	115.9 (4)
O6—La1—O5^ii^	63.19 (11)	C4—N8—La1	126.3 (3)
O3—La1—O5^ii^	124.85 (11)	C1—N8—La1	114.8 (3)
O1W—La1—O1	68.69 (11)	C7—O1—La1	122.9 (3)
O4^i^—La1—O1	122.78 (11)	C7—O1—La1^iii^	101.2 (3)
O6—La1—O1	151.51 (12)	La1—O1—La1^iii^	119.55 (13)
O3—La1—O1	71.74 (11)	C7—O2—La1^iii^	88.8 (3)
O5^ii^—La1—O1	108.11 (11)	C5—O3—La1	120.1 (3)
O1W—La1—O2W	130.93 (11)	C5—O4—La1^i^	120.6 (3)
O4^i^—La1—O2W	67.57 (12)	C6—O5—La1^ii^	121.0 (3)
O6—La1—O2W	72.86 (11)	C6—O6—La1	121.8 (3)
O3—La1—O2W	69.18 (11)	La1—O1W—H1W	113.1
O5^ii^—La1—O2W	123.82 (12)	La1—O1W—H2W	115.0
O1—La1—O2W	126.58 (11)	H1W—O1W—H2W	107.3
O1W—La1—O1^iii^	77.11 (11)	La1—O2W—H3W	111.1
O4^i^—La1—O1^iii^	132.53 (11)	La1—O2W—H4W	123.8
O6—La1—O1^iii^	114.65 (11)	H3W—O2W—H4W	106.5
O3—La1—O1^iii^	75.79 (11)		

Symmetry codes: (i) −*x*+1, −*y*+1, −*z*+1; (ii) −*x*, −*y*, −*z*; (iii) −*x*+1, −*y*, −*z*+1.

### Hydrogen-bond geometry (Å, °)

**Table:**

*D*—H···*A*	*D*—H	H···*A*	*D*···*A*	*D*—H···*A*
O1W—H1W···N1^iv^	0.84	1.97	2.796 (6)	170
O2W—H3W···O2^v^	0.84	1.94	2.737 (5)	157
O1W—H2W···O3^iii^	0.84	2.05	2.874 (5)	167
O2W—H4W···O6^vi^	0.84	2.09	2.825 (5)	146

Symmetry codes: (iv) *x*, *y*−1, *z*−1; (v) *x*, *y*, *z*−1; (iii) −*x*+1, −*y*, −*z*+1; (vi) −*x*+1, −*y*, −*z*.
